# SABinder: A Web Service for Predicting Streptavidin-Binding Peptides

**DOI:** 10.1155/2016/9175143

**Published:** 2016-08-17

**Authors:** Bifang He, Juanjuan Kang, Beibei Ru, Hui Ding, Peng Zhou, Jian Huang

**Affiliations:** ^1^Key Laboratory for Neuroinformation of Ministry of Education, School of Life Science and Technology, University of Electronic Science and Technology of China, Chengdu 610054, China; ^2^Center for Informational Biology, University of Electronic Science and Technology of China, Chengdu 610054, China

## Abstract

Streptavidin is sometimes used as the intended target to screen phage-displayed combinatorial peptide libraries for streptavidin-binding peptides (SBPs). More often in the biopanning system, however, streptavidin is just a commonly used anchoring molecule that can efficiently capture the biotinylated target. In this case, SBPs creeping into the biopanning results are not desired binders but target-unrelated peptides (TUP). Taking them as intended binders may mislead subsequent studies. Therefore, it is important to find if a peptide is likely to be an SBP when streptavidin is either the intended target or just the anchoring molecule. In this paper, we describe an SVM-based ensemble predictor called SABinder. It is the first predictor for SBP. The model was built with the feature of optimized dipeptide composition. It was observed that 89.20% (MCC = 0.78; AUC = 0.93; permutation test, *p* < 0.001) of peptides were correctly classified. As a web server, SABinder is freely accessible. The tool provides a highly efficient way to exclude potential SBP when they are TUP or to facilitate identification of possibly new SBP when they are the desired binders. In either case, it will be helpful and can benefit related scientific community.

## 1. Introduction

Phage display is a versatile technique to select peptides or proteins with specific affinity to a given target [1–4]. Unfortunately, target-unrelated peptides (TUP) are enriched in the biopanning results due to several intrinsic faults of phage libraries and panning systems [5, 6]. TUP can be divided into two types, propagation related and selection related. A propagation-related TUP can arise in the output of phage display because it has a propagation advantage [7, 8]. In contrary, a selection-related TUP can sneak into the results of biopanning as a result of reacting with other components of the biopanning systems instead of the target [6, 9]. As streptavidin (SA) is frequently used in phage display experiments [10–13], streptavidin-binding peptides (SBPs) repeatedly emerge in the biopanning results. Sometimes, SA is used as the intended target to find SBP [10, 11], which can be developed as affinity tags for protein purification and detection [14, 15]. More often in the biopanning system, however, SA is just a commonly used anchoring molecule that can efficiently capture the biotinylated target [12, 13]. In this case, SBPs that sneak into the biopanning results are selection-related TUP rather than desired binders [5]. They are discovered due to affinity to the capturing reagent SA instead of the target molecule. Taking them as intended binders may mislead subsequent studies. Therefore, it is important to know if a peptide is likely to be an SBP when SA exists in the biopanning system either as the intended target or as just the anchoring molecule.

Although the screening of phage-displayed random peptide libraries has become a key methodology for finding SBP, the wet-experimental technique is time-consuming and costly. With the increase of SBP, it is highly desirable to develop computational methods to identify SBP. Accordingly, it would be a feasible avenue to resort to the machine learning-based approaches, which have been proved to be quite powerful in dealing with protein and peptide classification problems [16–19]. Given this, a new method of classification for SBP by means of support vector machine (SVM) is proposed [20]. A computational tool for predicting SBP will certainly facilitate the cheaper and more rapid discovery of novel SBP.

In this paper, we describe SABinder, an ensemble SBP predictor based on support vector machine (SVM). It can be a helpful complement to the existing experimental measures such as subtractive selection and specific elution [6], which can reduce SBP in the biopanning results when SA is not the intended target. Besides, it can also be conducive to identify new SBP candidates when it aims to find new affinity tags.

## 2. Data and Methods

### 2.1. Datasets

**Figure 1:**
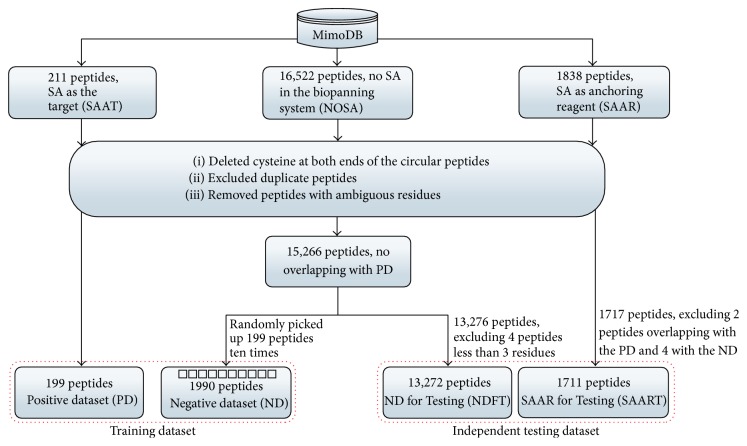
Flowchart of datasets construction. Training dataset and two independent testing datasets were constructed according to the above flowchart.

The datasets were collected from the BDB database [21–23], which aims to be an information portal to experimental results of biopanning. Our datasets from completely random combinatorial peptide libraries were acquired from MimoDB v4.0 released on September 30, 2013. Construction of all datasets is illustrated in Figure 1. The positive samples were from biopanning experiments in which SA is used as the intended target molecule. The negative samples were from biopanning experiments in which the target is anything else but not SA and SA does not exist in the biopanning system. The independent testing dataset is composed of two parts. One is collected from results of biopanning experiments where SA is used just as anchoring reagent. The other is from negative samples not used for training.

Both the training and the independent testing dataset were preprocessed as follows: (i) cysteine amino acids at both ends of the circular peptides were deleted; (ii) the duplicate sequences were eliminated; (iii) peptide sequences harboring ambiguous residues (“X”, “B,” and “Z”) or nonalpha characters were excluded. For the negative dataset, we also carefully compared each sequence with those sequences in the positive dataset and found there was no overlap between them. After a series of abovementioned processing, there were 1717 peptides in the independent testing dataset. In positive and negative dataset, there were 199 and 15,266 peptides, respectively. The negative samples remarkably outnumbered the positive samples. Therefore, downsampling strategy was proposed to work out the challenge by randomly picking out 199 peptides from the negative samples. To diminish random errors, such procedure was repeated ten times. The only one positive dataset with 199 peptides was paired with the ten negative subdatasets above, respectively. As a consequence, ten pairs of subdatasets were generated and each pair was made up of 199 peptides with specific affinity to SA and 199 peptides without affinity to SA. After picking out 1990 peptides as negative training dataset, we utilized the remaining 13,272 peptides (4 peptides less than 3 residues were excluded) in the negative samples for evaluation, which is called the Negative Dataset for Testing (NDFT) dataset. Besides we checked the first part of the independent testing dataset with 1717 peptides and found there were 2 peptides overlapping with the positive dataset and 4 with the negative training dataset. Therefore these six peptides were excluded and there were 1711 peptides left. We called it the SA as Anchoring Reagent for Testing (SAART) dataset. The number of positive and negative peptides in each dataset is listed in Table 1. The training dataset is provided in Supplementary Material A available online at http://dx.doi.org/10.1155/2016/9175143. And two independent testing datasets are provided in Supplementary Material B.

### 2.2. Features and Feature Selection

Extraction of a set of typical features is an extremely significant step in the process of pattern classification and has direct influence on the performance of the prediction model. For the sake of establishing the optimal prediction model, each peptide in the training dataset was encoded by 20 amino acid compositions (AACs) and 400 dipeptide compositions (DPCs), respectively. Definition of AAC and DPC was as the following equations:

(1) AACi=xi∑i=120xi,DPCj=yj∑j=1400yj,

where *i* stands for one of the 20 amino acids and *j* one of the 400 dipeptides. *x*(*i*) denotes the number of residues of each type and *y*(*j*) represents the number of dipeptides of each type in each sequence.

Feature selection technique was introduced to drop the irrelevant, redundant, and noisy features [24]. Its fundamental purpose is to enhance the efficiency and the degree of accuracy of the prediction model by seeking out the optimized feature. In this report, we implemented feature selection with AAC and DPC, respectively, to gain two sets of optimum features. The basic idea was characterized as follows: (i) the accuracy of each element was figured out; (ii) an element was put into an initially null set in descending order by accuracy one by one and the accuracy of each set was calculated when an element was added in; (iii) the set with the highest predictive accuracy was chosen as the optimal reduced subset. Ultimately the optimized AAC (OAAC) and the optimized DPC (ODPC) were obtained.

### 2.3. Support Vector Machine

The SVM has gained increasing popularity and also been extensively used in the field of bioinformatics [25–27]. It is a machine learning method which is based on the structural risk minimization (SRM) principle from statistical learning theory. In general, the principal idea of SVM is projecting the input vectors into a high-dimensional space with the kernel function, and a maximized margin separation hyperplane is constructed in the transformed space. In this work, our prediction assignment performed by SVM was considered to be a binary classification problem. And the SVM model was developed by using the software LibSVM3.11 [28], which is an integrated software for support vector classification and can be downloaded free of charge from http://www.csie.ntu.edu.tw/~cjlin/libsvm/. Generally, four kinds of kernel functions, that is, polynomial function, linear function, radial basis function (RBF), and sigmoid function, are available to implement prediction. Since preliminary trial indicated that the RBF achieved the highest predictive accuracy, we utilized the RBF kernel function in the current work. In addition, optimization of the kernel width parameter *g* and the regularization parameter *c* was via the grid search approach.

### 2.4. Prediction Assessment

In this study all models constructed were evaluated by using fivefold cross-validation, where the whole dataset is split into five groups at random, each containing equal number of peptides. Four groups are used for training and the remaining one is used for testing. This process is repeated five times. In such a way, each group is used as the test group once. Eventually the average prediction accuracy of five kinds of combination is calculated as the final accuracy of one model. For assessing the performance of the model, we used four common parameters, namely, sensitivity (Sn), specificity (Sp), accuracy (Acc), and Matthews correlation coefficient (MCC). The following equations were used to compute these parameters:

(2) Sn=TPTP+FN,Sp=TNFP+TN,Acc=TP+TNTP+FN+FP+TN,MCC=TP×TN−FP×FNTP+FPTP+FNTN+FPTN+FN.

In the above formulas, TP and TN are the number of correctly predicted SBPs and non-SBPs, respectively. Accordingly FP and FN represent the number of wrongly predicted SBPs and non-SBPs, respectively. MCC is one of the most robust parameters in any class predictive approach. An MCC equal to 1 is deemed to be the best prediction, whereas 0 is for a completely random prediction and −1 is an absolutely adverse prediction. In addition, the competence of the model is illustrated with the Receiver Operating Characteristic (ROC) curve. The area under the ROC curve (AUC) is used as the performance measure. For a perfect prediction, the maximum value of the AUC equals 1.0. For a random guess, the AUC equals 0.5.

To evaluate the statistical significance of the observed classification accuracy, a permutation test with 1000 permutations was conducted by shuffling the labels of the dataset [20]. Then fivefold cross-validation was performed against the label-permuted dataset. For each permutation trial, an Acc_perm can be obtained. The final *p* value was computed by the number of times that Acc_perm was larger than the observed classification accuracy based on the original dataset divided by the total permutation times. The *p* value less than 0.05 was considered statistically significant.

### 2.5. Construction of the SVM Models

The prediction models based on SVM were built with AAC, OAAC, DPC, and ODPC by using the ten pairs of the subdatasets, respectively. To reduce errors resulting from an individual predictive model, the voting strategy was proposed to construct an ensemble predictor. The SABinder predictor was established with ODPC. Each peptide input was subjected to the prediction of ten submodels separately [24]. Each submodel will compute the peptide's possibility of being an SBP. The final probability was calculated by averaging the results of ten submodels. If the value is equal to or greater than the threshold, the peptide will be identified as an SBP. The threshold to distinguish between predicted positives and negatives (tp) ranges from 0 to 1. However, it is set to 0.5 by default. That is to say, a peptide will be predicted to be a streptavidin-binding peptide (SBP) if the probability is 0.5 or higher. Users can adjust the threshold according to their own needs. For instance, users should set a lower tp if they do not care about false positives and just want to exclude all possible SBPs. On the contrary, users should increase the tp to 0.95, for example, if their purpose is to discover some novel SBPs with greater confidence. We also provided the voting results, which represented how many submodels voted the peptide as an SBP. The higher the number, the higher the reliability that the peptide is an SBP.

### 2.6. Evaluation on Independent Testing Datasets

To assess the performance of our model in a rigorous way, SABinder was evaluated on independent testing datasets. In this report, we constructed two independent testing datasets: one consisting of 13,272 peptides was the NDFT dataset and the other one containing 1711 peptides was the SAART dataset. The two independent datasets were predicted by SABinder. Furthermore, a chi-square test was utilized to determine whether the estimated positive rate from the SAART dataset was significantly higher than that from the NDFT dataset.

## 3. Results and Discussion

### 3.1. Performances of SVM-Based Models Trained with Different Features

Four models on the base of SVM were established with features, that is, AAC, OAAC, DPC, and ODPC, respectively, in the current work. Fivefold cross-validation was applied to evaluate the effectiveness of SVM-based models. Performances of each submodel are provided in Table S1 in Supplementary Material C. The standard deviations of classification results and their average performances are shown in Table 2. It has been verified that DPC-based methods are superior to AAC-based methods in classification of proteins [29]. As would be expected, the DPC-based classifier reached not only much higher accuracy about 85% but also better MCC around 0.71. Moreover, OAAC and ODPC features are selected through a procedure which has been described and used in peptide classification [24]. In this work, two sets of optimum features, namely, OAAC and ODPC, were obtained through feature selection against AAC and DPC, respectively. As was previously known, HPQ is the most common SA-binding motif [30]. Amino acids H, P, and Q do appear in the set of OAAC. However in the set of ODPC, besides HP and PQ, amino acid pairs such as PP, LP, PL, PS, SP, and TP also appear. These findings may advance the discovery of new SA-binding motifs. In addition, the model built with ODPC attains the maximum accuracy around 89% and an impressive MCC about 0.78. This demonstrates that the combination of feature encoding scheme and feature selection technique can achieve preferable predictive performance.

### 3.2. Prediction Performances of Various Machine Learning Methods

To find the perfect machine learning method, we tried various classical machine learning methods against ODPC. The top SVM model was compared with five other state-of-the-art classifiers, namely, Naïve Bayes, Logistic Function, RBF network, Decision Tree J48, and Random Forest, implemented in WEKA [31]. As fivefold cross-validation results shown in Table 3, the average accuracy of the SVM model is approximately 11%, 17%, 11%, 7%, and 3% higher than Naïve Bayes, Logistic Function, RBF network, Decision Tree J48, and Random Forest classifiers, respectively. This reveals that the SVM-based method performs best when comparing with other machine learning methods. Results of each submodel are provided in Table S2 in Supplementary Material C.

### 3.3. Constructing an Integrated Predictor

**Figure 2:**
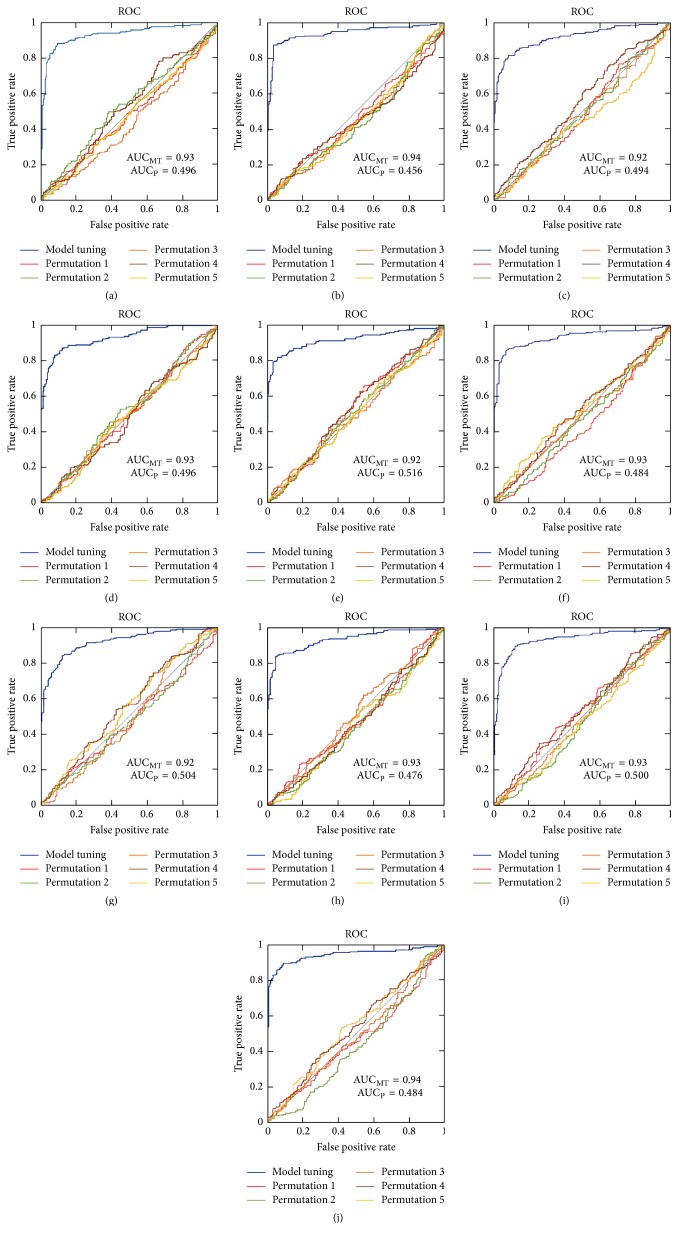
ROC curves for model tuning and five permutations. AUC_MT_ and AUC_P_ represent AUC for model tuning and average AUC for five permutations, respectively. For all 10 submodels, the AUC of model tuning is much higher than the permutated ones, which shows an excellent prediction. For visualization, only five ROC curves for five out of 1000 permutations were plotted.

Finally, we trained the SVM-based model on ten pairs of subdatasets with ODPC. The voting strategy was offered to construct a holistic predictor to mitigate errors induced by any single predictive model. Fivefold cross-validation results showed that the highest accuracy of 89.20% was attained with 0.78 MCC, 84.72% sensitivity, and 93.67% specificity. As shown in Figure 2, the ROC curves for ten submodels tuning were compared with five ROC curves for five permutations. And the average AUC for model tuning is approximately 0.93, which shows an excellent prediction. The permutation test resulted in a *p* value of <0.001. Accordingly the SVM-based predictor built with ODPC was implemented into an online web service, called SABinder. The common gateway interface script for SABinder was written using Perl. SABinder is freely available at http://i.uestc.edu.cn/sarotup/cgi-bin/SABinder.pl. The web service allows user to feed the peptide sequence in FASTA format or as plain text. After submission, the prediction result will be returned and displayed in a table.

### 3.4. Evaluation of SABinder

To evaluate SABinder, we constructed two independent testing datasets. One is the NDFT dataset with 13,272 non-SA binders. They are taken from the negative dataset. However, they are not used for model building. The other is called the SAART dataset which contains 1711 peptides. Each peptide in the NDFT dataset should have a lower possibility to be an SBP, since SA are not used in corresponding experiments. Peptides in the SAART dataset may have a higher possibility to be SBP, since SA are used in corresponding experiments though not as targets. SABinder was tested on the two independent datasets. Results from the NDFT dataset showed that 1169 peptides were predicted to be possible SA binders with a positive rate of 8.81% when tp was set to 0.5. Indeed, results from the SAART dataset showed a positive rate of 12.16% (208 of the 1711 peptides were predicted to be SBP), which is significantly higher than the results from the NDFT dataset (*p* < 0.05, chi-square test). In addition, we also did the above analysis when tp was set to 0.3 and 0.7. It was observed that the positive rate in the SAART dataset was statistically greater than that in the NDFT dataset in both cases.

### 3.5. Comparison between SABinder and the Existing Tools

We developed an SVM-based ensemble predictor with ODPC for detecting SBP in the current study. Fivefold cross-validation results indicated that our prediction method gave an efficient and powerful performance. In our previous work, SAROTUP, a suite of web tools capable of scanning, reporting, and excluding potential target-unrelated peptides from biopanning results [30], was developed. There are 5 SA-binding motifs, namely, HPQ, EPDW(F/Y), DVEAW(L/I), GD(F/W)XF, and PWXWL, in TUPScan [30]. Any peptides matching these five motifs are likely to be SA binders. With regard to those sequences which cannot match motifs, a search of MimoDB (renamed BDB) and PepBank is recommended to find out whether these sequences are screened out in other experiments with various targets [21, 22, 32, 33]. If so, they are probably peptides binding to unintended materials such as SA. Also MimoBlast is proposed to check if there are peptides in the MimoDB database that are identical or similar to the peptides user submitted. Highly similar peptides obtained with various targets might also be TUP. For those peptides which can neither match known SA-binding motifs nor be found in databases, SABinder is currently the only and the best choice.

## 4. Conclusions

In this report, we have developed an SVM-based ensemble predictor with ODPC for detecting SBP. Fivefold cross-validation was used to assess the performance of the model. Comparing with other machine learning methods, the SVM-based model was the best-performing predictor and a maximum accuracy of 89.20% was achieved with 0.78 MCC, 84.72% sensitivity, and 93.67% specificity, respectively. In the end, the SVM-based model was implemented into an online web service called SABinder, which is freely available at http://i.uestc.edu.cn/sarotup/cgi-bin/SABinder.pl. On one hand, the tool offers a highly efficient way to exclude SA binders when they are TUP; on the other hand, it contributes to the identification of novel SBP when they are desired binders and will facilitate the development of related products.

## Supplementary Material

Supplementary Material A: All peptides in the positive dataset and the negative dataset are provided. Supplementary Material B: All peptides in the NDFT and SAART independent testing datasets are provided. Supplementary Material C: Performances of each submodel are provided in Table S1. Results of each submodel trained with various machine learning methods are provided in Table S2.

## Figures and Tables

**Table 1:** Number of positive and negative peptides in each dataset.

Dataset	Number of positive peptides	Number of negative peptides	Length distribution (mean ± std)
Training dataset	199	1990	9 ± 3.49
NDFT dataset	0	13272	9 ± 3.24
SAART dataset^*∗*^	—	—	10 ± 4.18

^*∗*^SAART dataset: the numbers of positive peptides and negative peptides are not determined.

**Table 2:** Performances of SVM-based models trained with different features.

Feature	Sn (%) (mean ± std)	Sp (%) (mean ± std)	Acc (%) (mean ± std)	MCC (mean ± std)
Amino acid composition (AAC)	79.35 ± 1.96	78.79 ± 2.65	79.07 ± 1.75	0.58 ± 0.04
Optimized amino acid composition (OAAC)	78.14 ± 3.9	82.31 ± 4.45	80.23 ± 1.42	0.61 ± 0.03
Dipeptide composition (DPC)	79.14 ± 3.50	91.26 ± 1.92	85.20 ± 1.40	0.71 ± 0.03
Optimized dipeptide composition (ODPC)	***84.72 ± 2.19***	***93.67 ± 1.87***	***89.20 ± 1.23***	***0.79 ± 0.02***

Std: standard deviation.

**Table 3:** The prediction performances of various machine learning methods.

Machine learning methods	Sn (%) (mean ± std)	Sp (%) (mean ± std)	Acc (%) (mean ± std)	MCC (mean ± std)
Support vector machine	***84.72 ± 2.19***	***93.67 ± 1.87***	***89.20 ± 1.23***	***0.79 ± 0.02***
Naïve Bayes	78.85 ± 3.90	77.40 ± 1.73	78.11 ± 2.47	0.56 ± 0.05
Random Forest	84.80 ± 2.30	88.00 ± 5.22	86.41 ± 2.41	0.73 ± 0.05
Decision Tree J48	76.90 ± 1.10	88.24 ± 4.31	82.57 ± 2.00	0.66 ± 0.04
RBF network	79.00 ± 4.18	78.50 ± 2.33	78.74 ± 2.57	0.58 ± 0.05
Logistic Function	76.40 ± 3.22	67.83 ± 3.82	72.11 ± 3.23	0.44 ± 0.06

Std: standard deviation.
