# Supplementary material for: SABinder: A Web Service for Predicting Streptavidin-Binding Peptides
Source: Biomed Res Int. 2016 Aug 17;2016:9175143. doi: 10.1155/2016/9175143 (PMC5005764; doi:10.1155/2016/9175143)
Supplement: Supplementary file 1 — Supplementary Material A: All peptides in the positive dataset and the negative dataset are provided. Supplementary Material B: All peptides in the NDFT and SAART independent testing datasets are provided. Supplementary Material C: Performances of each submodel are provided in Table S1. Results of each submodel trained with various machine learning methods are provided in Table S2. [file 9175143.f1.zip › Supplementary material C.docx]

**SUPPLEMENTARY MATERIALS**

**Table S1** Performances of SVM-based submodels trained with different features

| **Features** | **Submodels** | **Sn (%)** | **Sp (%)** | **Acc (%)** | **MCC** |
| --- | --- | --- | --- | --- | --- |
| Amino acid  component (AAC) | 1 | 77.39 | 76.38 | 76.88 | 0.538 |
|  | 2 | 82.41 | 75.88 | 79.15 | 0.584 |
|  | 3 | 78.89 | 78.89 | 78.89 | 0.578 |
|  | 4 | 78.89 | 80.9 | 79.9 | 0.598 |
|  | 5 | 75.88 | 77.39 | 76.63 | 0.533 |
|  | 6 | 80.4 | 84.92 | 82.66 | 0.654 |
|  | 7 | 81.41 | 77.39 | 79.4 | 0.588 |
|  | 8 | 78.39 | 77.39 | 77.89 | 0.558 |
|  | 9 | 78.89 | 78.89 | 78.89 | 0.578 |
|  | 10 | 80.9 | 79.9 | 80.4 | 0.608 |
| Optimized amino acid composition (OAAC) | 1 | 74.37 | 82.91 | 78.64 | 0.575 |
|  | 2 | 81.91 | 79.4 | 80.65 | 0.613 |
|  | 3 | 75.88 | 85.43 | 80.65 | 0.616 |
|  | 4 | 80.9 | 79.4 | 80.15 | 0.603 |
|  | 5 | 76.38 | 78.89 | 77.64 | 0.553 |
|  | 6 | 79.9 | 85.93 | 82.91 | 0.659 |
|  | 7 | 81.41 | 79.9 | 80.65 | 0.613 |
|  | 8 | 80.4 | 79.9 | 80.15 | 0.603 |
|  | 9 | 80.4 | 78.89 | 79.65 | 0.593 |
|  | 10 | 69.85 | 92.46 | 81.16 | 0.64 |
| Dipeptide composition  (DPC) | 1 | 78.89 | 90.45 | 84.67 | 0.698 |
|  | 2 | 82.91 | 91.96 | 87.44 | 0.752 |
|  | 3 | 78.39 | 89.45 | 83.92 | 0.683 |
|  | 4 | 80.9 | 90.45 | 85.68 | 0.717 |
|  | 5 | 77.39 | 90.45 | 83.92 | 0.684 |
|  | 6 | 80.9 | 90.45 | 85.68 | 0.717 |
|  | 7 | 70.35 | 95.98 | 83.17 | 0.686 |
|  | 8 | 79.9 | 89.45 | 84.67 | 0.697 |
|  | 9 | 79.9 | 91.46 | 85.68 | 0.718 |
|  | 10 | 81.91 | 92.46 | 87.19 | 0.748 |
| Optimized dipeptide  composition (ODPC) | 1 | 85.93 | 92.96 | 89.45 | 0.791 |
|  | 2 | 86.93 | 94.97 | 90.95 | 0.822 |
|  | 3 | 84.42 | 91.46 | 87.94 | 0.761 |
|  | 4 | 83.42 | 93.97 | 88.69 | 0.778 |
|  | 5 | 80.4 | 95.98 | 88.19 | 0.773 |
|  | 6 | 86.93 | 94.47 | 90.7 | 0.816 |
|  | 7 | 83.92 | 90.45 | 87.19 | 0.745 |
|  | 8 | 82.91 | 94.97 | 88.94 | 0.785 |
|  | 9 | 87.44 | 91.96 | 89.7 | 0.795 |
|  | 10 | 84.92 | 95.48 | 90.2 | 0.809 |

**Table S2** Prediction performances of submodels trained with various machine learning methods

| **Machine learning methods** | **Submodels** | **Sn (%)** | **Sp (%)** | **Acc (%)** | **MCC** |
| --- | --- | --- | --- | --- | --- |
| SVM | 1 | 85.93 | 92.96 | 89.45 | 0.791 |
|  | 2 | 86.93 | 94.97 | 90.95 | 0.822 |
|  | 3 | 84.42 | 91.46 | 87.94 | 0.761 |
|  | 4 | 83.42 | 93.97 | 88.69 | 0.778 |
|  | 5 | 80.4 | 95.98 | 88.19 | 0.773 |
|  | 6 | 86.93 | 94.47 | 90.7 | 0.816 |
|  | 7 | 83.92 | 90.45 | 87.19 | 0.745 |
|  | 8 | 82.91 | 94.97 | 88.94 | 0.785 |
|  | 9 | 87.44 | 91.96 | 89.7 | 0.795 |
|  | 10 | 84.92 | 95.48 | 90.2 | 0.809 |
| Naïve Bayes | 1 | 81.4 | 78.9 | 80.2 | 0.603 |
|  | 2 | 82.9 | 79.4 | 81.2 | 0.624 |
|  | 3 | 74.9 | 76.4 | 75.6 | 0.513 |
|  | 4 | 74.9 | 76.4 | 75.6 | 0.513 |
|  | 5 | 76.9 | 74.4 | 75.6 | 0.513 |
|  | 6 | 81.4 | 75.9 | 78.6 | 0.574 |
|  | 7 | 73.9 | 76.9 | 75.4 | 0.508 |
|  | 8 | 85.4 | 77.4 | 81.4 | 0.63 |
|  | 9 | 79.9 | 79.9 | 79.9 | 0.598 |
|  | 10 | 76.9 | 78.4 | 77.6 | 0.553 |
| Random Forest | 1 | 87.9 | 80.4 | 84.2 | 0.685 |
|  | 2 | 85.9 | 89.4 | 87.7 | 0.754 |
|  | 3 | 80.4 | 87.4 | 83.9 | 0.68 |
|  | 4 | 83.4 | 87.9 | 85.7 | 0.714 |
|  | 5 | 85.4 | 79.9 | 82.7 | 0.654 |
|  | 6 | 83.9 | 94.5 | 89.2 | 0.788 |
|  | 7 | 82.4 | 92.5 | 87.4 | 0.753 |
|  | 8 | 86.9 | 83.4 | 85.2 | 0.704 |
|  | 9 | 86.9 | 92.5 | 89.7 | 0.795 |
|  | 10 | 84.9 | 92 | 88.4 | 0.771 |
| Decision Tree J48 | 1 | 76.4 | 91.5 | 83.9 | 0.686 |
|  | 2 | 77.9 | 78.4 | 78.1 | 0.563 |
|  | 3 | 77.4 | 85.4 | 81.4 | 0.63 |
|  | 4 | 76.9 | 92.5 | 84.7 | 0.702 |
|  | 5 | 74.9 | 89.9 | 82.4 | 0.656 |
|  | 6 | 76.9 | 91 | 83.9 | 0.685 |
|  | 7 | 76.9 | 85.4 | 81.2 | 0.625 |
|  | 8 | 77.9 | 91.5 | 84.7 | 0.7 |
|  | 9 | 78.4 | 86.9 | 82.7 | 0.656 |
|  | 10 | 75.4 | 89.9 | 82.7 | 0.66 |
| RBF network | 1 | 82.4 | 77.9 | 80.2 | 0.605 |
|  | 2 | 85.4 | 78.4 | 81.9 | 0.64 |
|  | 3 | 75.4 | 78.9 | 77.1 | 0.543 |
|  | 4 | 77.9 | 79.9 | 78.9 | 0.578 |
|  | 5 | 76.4 | 72.9 | 74.6 | 0.493 |
|  | 6 | 80.4 | 81.4 | 80.9 | 0.618 |
|  | 7 | 70.9 | 77.9 | 74.4 | 0.489 |
|  | 8 | 82.9 | 77.9 | 80.4 | 0.609 |
|  | 9 | 78.4 | 80.9 | 79.6 | 0.593 |
|  | 10 | 79.9 | 78.9 | 79.4 | 0.588 |
| Logistic Function | 1 | 72.4 | 65.8 | 69.1 | 0.383 |
|  | 2 | 77.9 | 70.9 | 74.4 | 0.489 |
|  | 3 | 79.9 | 70.4 | 75.1 | 0.505 |
|  | 4 | 72.4 | 69.8 | 71.1 | 0.422 |
|  | 5 | 71.4 | 59.8 | 65.6 | 0.314 |
|  | 6 | 78.9 | 67.8 | 73.4 | 0.47 |
|  | 7 | 79.9 | 72.9 | 76.4 | 0.529 |
|  | 8 | 75.9 | 63.8 | 69.8 | 0.4 |
|  | 9 | 77.9 | 68.3 | 73.1 | 0.464 |
|  | 10 | 77.4 | 68.8 | 73.1 | 0.464 |
